# Caffeic Acid Derivatives Inhibit the Growth of Colon Cancer: Involvement of the PI3-K/Akt and AMPK Signaling Pathways

**DOI:** 10.1371/journal.pone.0099631

**Published:** 2014-06-24

**Authors:** En-Pei Isabel Chiang, Shu-Yao Tsai, Yueh-Hsiung Kuo, Man-Hui Pai, Hsi-Lin Chiu, Raymond L. Rodriguez, Feng-Yao Tang

**Affiliations:** 1 Department of Food Science and Biotechnology, National Chung Hsing University, Taichung, Taiwan, Republic of China; 2 NCHU-UCD Plant and Food Biotechnology Program and Agricultural Biotechnology Center, National Chung Hsing University, Taichung, Taiwan, Republic of China; 3 Department of Health and Nutrition Biotechnology, Asia University, Taichung, Taiwan, Republic of China; 4 Department of Chinese Pharmaceutical Sciences and Chinese Medicine Resources, China Medical University, Taichung, Taiwan, Republic of China; 5 Department of Biotechnology, Asia University, Taichung, Taiwan, Republic of China; 6 Department of Anatomy, Taipei Medical University, Taipei, Taiwan, Republic of China; 7 Department of Molecular and Cellular Biology, University of California Davis, Davis, California, United States of America; 8 Biomedical Science Laboratory, Department of Nutrition, China Medical University, Taichung, Taiwan, Republic of China; National Health Research Institutes, Taiwan

## Abstract

**Background:**

The aberrant regulation of phosphatidylinositide 3-kinases (PI3-K)/Akt, AMP-activated protein kinase (AMPK) and mammalian target of rapamycin (m-TOR) signaling pathways in cancer has prompted significant interest in the suppression of these pathways to treat cancer. Caffeic acid (CA) has been reported to possess important anti-inflammatory actions. However, the molecular mechanisms by which CA derivatives including caffeic acid phenethyl ester (CAPE) and caffeic acid phenylpropyl ester (CAPPE), exert inhibitory effects on the proliferation of human colorectal cancer (CRC) cells have yet to be elucidated.

**Methodology/Principal Findings:**

CAPE and CAPPE were evaluated for their ability to modulate these signaling pathways and suppress the proliferation of CRC cells both *in vitro* and *in vivo*. Anti-cancer effects of these CA derivatives were measured by using proliferation assays, cell cycle analysis, western blotting assay, reporter gene assay and immunohistochemical (IHC) staining assays both *in vitro* and *in vivo*. This study demonstrates that CAPE and CAPPE exhibit a dose-dependent inhibition of proliferation and survival of CRC cells through the induction of G_0_/G_1_ cell cycle arrest and augmentation of apoptotic pathways. Consumption of CAPE and CAPPE significantly inhibited the growth of colorectal tumors in a mouse xenograft model. The mechanisms of action included a modulation of PI3-K/Akt, AMPK and m-TOR signaling cascades both *in vitro* and *in vivo*. In conclusion, the results demonstrate novel anti-cancer mechanisms of CA derivatives against the growth of human CRC cells.

**Conclusions:**

CA derivatives are potent anti-cancer agents that augment AMPK activation and promote apoptosis in human CRC cells. The structure of CA derivatives can be used for the rational design of novel inhibitors that target human CRC cells.

## Introduction

Colorectal cancer (CRC) is one of the leading causes of cancer and cancer mortality in many countries [1], [2]. In the United States alone, approximately 50,000 deaths are attributed to this cancer annually [1], [2]. Many studies have indicated that mutations of the phosphatidylinositide 3-kinase (PI3-K)/Akt and mitogen-activated protein kinase (MAPK)/extracellular-signal-regulated kinase (ERK) molecules are commonly observed in various types of cancer [3], [4]. For example, oncogenic activation of PI3-K/Akt molecules enhances cell proliferation by increasing the cyclin D1 level [5], [6]. It is well known that the aberrant expression of the cyclin D1 and Cdk4 proteins is involved in the proliferation of CRC cells [7]. Suppression of the PI3-K/Akt and MAPK/ERK signaling pathways leads to the blockade of cell proliferation and demonstrates the importance of these signaling cascades in the control of both cell cycle progression and cell growth during cancer development [4], [8]. Therefore, the PI3-K/Akt and MAPK/ERK signaling pathways play predominant roles in determining the fate of tumor growth. Malignant cancer cells detach from the primary tumor and migrate across structural barriers, including basement membranes and the surrounding stromal extracellular matrix (ECM) [9]. Tumor invasion and metastasis both require an increase in the expression of matrix metalloproteinases (MMPs) and the degradation of ECM [9], [10]. MMPs are zinc-dependent endopeptidases capable of degrading ECM components [11]. Enzymes such as MMP-9 degrade ECM and create a microenvironment that maintains tumor development [10], [11].

AMP-activated protein kinase (AMPK) is a fuel-sensing molecule that functions as a regulator of energy balance [12]. AMPK has been shown to be ubiquitously expressed in mammalian cells and to be involved in energy homeostasis [13]. An increased adenosine monophosphate (AMP)/adenosine triphosphate (ATP) ratio, reflecting a decrease in the cell's energy state, leads to the activation of the AMPK protein by phosphorylation [14]. The augmentation of AMPK activation is thought to be inversely correlated with cancer risk [15]. Recent studies have further suggested that the activation of the PI3-K/Akt and MAPK/ERK signaling molecules is associated with a decreased level of phosphorylated (activated) AMPK in the course of tumor progression [16], [17]. Additional studies concluded that AMPK agonists are effective in the treatment of cancer [15], [18], while other studies showed that the lipogenic enzyme fatty acid synthase (FASN) is regulated by energy intake and plays a crucial role in carcinogenesis [19]. One recent study reported that FASN expression is correlated with the growth and progression of CRC [20]. The phosphorylation (i.e. activation) of Akt was shown to induce the expression of FASN and to trigger aggressive malignancy in cancer cells [21]. In contrast, treatment with an AMPK agonist, leading to the activation of AMPK, suppressed the expression of FASN and blocked the growth of colorectal tumor [22]–[24]. Moreover, epidemiological studies further indicated that AMPK (PRKAG2) single-nucleotide polymorphism (SNP) is associated with risk of human CRC [25]. Thus, AMPK-mediated energy homeostasis has attracted interest in this pathway as a means of treating human colon cancer.

Many studies have demonstrated that phenolic acid compounds function as potent antioxidants [26]. Among them, caffeic acid (CA) is a non-vitamin phenolic compound found largely in vegetables and fruit. In addition to its antioxidant activity, CA exerts anti-inflammatory effects in several kinds of cells [27], [28]. Recent studies indicated that caffeic acid phenethyl ester (CAPE), a CA derivative naturally isolated from honeybee propolis, also exerts its beneficial effects through antioxidant and anti-inflammatory activities [29], [30]. Furthermore, it has been demonstrated that CAPE inhibits the proliferation of cancer cells and act as a potential anti-cancer agent [31], [32]. However, there is no report of the inhibitory effects of CA derivatives on the AMPK pathway and/or FASN expression during the progression of CRC. Moreover, the lack of consistent results across numerous studies and the failure to determine the mechanism of action of the CA derivatives may explain the difficulty in demonstrating the *in vivo* benefits of CA derivative supplementation against CRC. We investigated, therefore, the inhibitory effects of various CA derivatives on human CRC cells both *in vitro* and *in vivo*. The results demonstrated that CA derivatives such as CAPE and caffeic acid phenylpropyl ester (CAPPE) significantly inhibited cellular proliferation in human CRC cells. CAPE and CAPPE induced cell cycle arrest through the suppression of the PI3-K/Akt and mTOR signaling pathways. Furthermore, CA derivatives reduced cellular ATP levels and suppressed FASN expression. The mechanism of action was associated in part with an augmentation of the AMPK pathway. The results of this study suggest that CA derivatives act as chemopreventive agents against human CRC by modulating the PI3-K/Akt, mTOR and AMPK signaling pathways both *in vitro* and *in vivo*.

## Materials and Methods

### Reagents and antibodies

**Figure 1:**
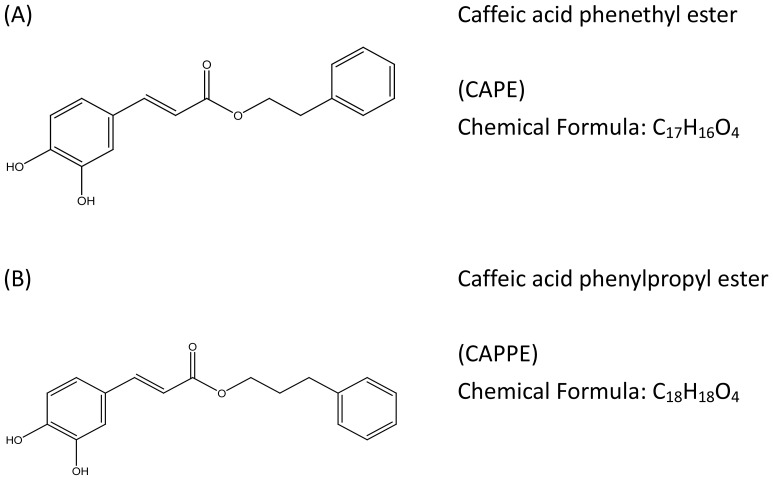
Chemical structure of the CA derivatives. The CA derivatives are depicted in Fig. 1. (A) CAPE and (B) CAPPE differ in the elongation of the alkyl side chain of the caffeic acid ester.

Human colon cancer cells HCT-116 and SW-480 were purchased from American Type Culture Collection (Walkersville, MD). The following monoclonal antibodies were purchased from Cell Signaling Technology, Inc.: Anti- N-cadherin (#4061), PTEN (#9559), anti-phosphorylation PDK1 (Ser241; #3061), total-PDK1(#3062), anti-phosphorylation Akt (S473; #4060), total-Akt (#9272), anti-phosphorylation GSK3α (S21; #9327), total- GSK3α (4337), anti-phosphorylation GSK3β (S9; #9323), total- GSK3β (#9315), anti-phosphorylation FOXO3 (T32; #9464), total- FOXO3 (#12829), total- TSC1 (#6935), total- TSC2 (#3990), total- LKB1 (#3047), total- 14-3-3 (#8312),anti-phosphorylation ERK 1/2 (T202/Y204; #9101), total-ERK 1/2 (#9102), anti-phosphorylation AMPKα (T172; #2535), total-AMPKα (#5832), anti-phosphorylation m-TOR (S2448; #5536), total-m-TOR (2983), anti-FASN(#3180), anti-NF-κB (p65) (#3033), anti-Cdk4(#2906), anti-p21^waf/cip1^(#2947), anti-cyclin E(#4132), anti-cyclin D1(#2978), anti-c-myc (#9402) and anti-Lamin A (#2032) (Danvers, MA). The anti- β-actin (# A2066) antibody and compound C (specific inhibitor of AMPK) were purchased from Sigma (St Louis, MO). The active Akt (Myr-Akt1, Addgene plasmid # 9008) and control empty vector (pcDNA3, Addgene plasmid # 10792) were obtained from Addgene. The tumor necrosis factor- α (TNF-α) recombinant protein was from R&D System (Minneapolis, MN). The nuclear Protein Extract Reagent Kit was purchased from Pierce Biotechnology Inc. (Lackford, IL). The luminescence ATP detection assay kit (ATPlite kit) was purchased from Perkin Elmer Life Science (Boston, MA). The NF-κB response element (NF-κB-RE) plasmid and Dual-Luciferase Reporter Assay kit were purchased from Promega (Madison, WI). PI (propidium Iodine) and anti- proliferating cell nuclear antigen (PCNA) (#610664) monoclonal antibodies were purchased from BD Biosciences Inc. (Franklin Lakes, NJ). CA derivatives, including CAPE and CAPPE (Figure 1) were provided by Dr. Y. H. Kuo (China Medical University). These CA derivatives were dissolved in dimethyl sulfoxide (DMSO) at a concentration of 200 mM stock solution and stored at −20°C. Immediately before the experiment, the stock solution was added to the cell culture medium, as described previously.

### Cell culture

Briefly, human CRC cells were cultured in a 37°C humidified incubator with 5% CO_2_ and grown to confluency using fetal bovine serum (FBS) supplemented RPMI-1640 media. The cells used in the different experiments have the same passage number. RPMI-1640 medium was supplemented with 10% heat-inactivated FBS, 2 mM L-glutamine and 1.5 g/L sodium bicarbonate.

### Supplementation with CA derivatives

Human CRC cells were incubated with different concentrations (0, 5, 10, 20, 50, and 100 µM) of the CA derivatives for 2 h or 24 h. For efficient uptake of the CA derivatives by human colon cancer cells, these compounds were incorporated into FBS for 30 min and mixed with the medium. In control groups, cells were incubated with an equivalent volume of solvent DMSO (final concentration: 0.05% v/v) as a carrier vehicle.

### Assessment of cell proliferation

The MTT (3-[4,5-dimethhylthiaoly]- 2,5-diphenyltetrazolium bromide) assay was conducted to detect the cell proliferation. Human CRC cells were seeded in 24-well plates, each well containing 1×10^5^ cells. After 24 h, the culture medium was replaced by media containing CA derivatives at one of five concentrations (i.e.,0, 5, 10, 20, 50 and 100 µM) in the presence or absence of compound C. Transfections of constitutively active Akt (Myr-Akt1, Addgene plasmid 9008) and empty vector (pcDNA3, Addgene plasmid 10792) were conducted by using Lipofectamine LTX transfection reagent. Each concentration was tested in triplicate. At the end of the experiment, one of the plates was taken out and fresh MTT (final concentration 0.5 mg/mL in PBS) was added to each well. After 2 hr incubation, the culture media were discarded, 200 µL of acidic isopropanol were added to each well and vibrated to dissolve the depositor. The optical density was measured at 570 nm with a microplate reader.

### Quantitative analysis of cell cycle by flow cytometry

Human colon cancer CRC cells were cultured into 6-well plates at a density of 1×10^6^ cells per well. Before the experiment, cells were synchronized by culturing them in 0.05% FBS supplemented RPMI-1640 media overnight until CAPE or CAPPE treatment. To measure the distribution of the cell cycle, cells were treated with CAPE or CAPPE (0, 10, 50, 100 µM) for an additional 24 h. Cells were harvested after treatment with a solution of trypsin and ethylenediaminetetraacetic acid (EDTA) and suspended with the binding buffer (1×10^5^ cells/mL). Human CRC cells were stained with PI and analyzed following the manufacturer's protocol. Briefly, five microliters of PI were added to the suspended cells and incubated at room temperature in the dark and analyzed by BD FACSCanto flow cytometry (BD Biosciences Inc., Franklin Lakes, NJ). The PI-stained cells were analyzed using accessory software.

### Xenograft implanation of tumor cells

To establish the mouse xenograft model, subconfluent cultures of colon cancer HCT-116 cells were given fresh medium 24 h before being harvested by a brief treatment with 0.25% trypsin and 0.02% EDTA. Trypsinization was stopped with medium containing 10% FBS, and the cells were washed twice and resuspended in serum-free RPMI 1640 medium. Only single-cell suspensions with a viability of >90% were used for the injections.

### Animals, Diet and CA Derivative Supplementation

Adult (3–4 week old) BALB/C AnN-Foxn1 nude mice (19–22 g) were obtained from the National Laboratory Animal Center (Taipei, Taiwan). Mice were maintained under specific pathogen-free conditions in facilities approved by the National Laboratory Animal Center in accordance with current regulations and standards (animal protocol no. 102-142-N). The animal use protocol listed above has been reviewed and approved by the Institutional Animal Care and Use Committee at China Medical University. The animal study was conducted according to the national guideline and the approved animal protocol in order to maintain animal welfare and ameliorate suffering in the experimental animals. During the entire experimental period, mice were fed a standard Lab 5010 Diet purchased from LabDiet Inc. (St. Louis, MO, USA). The standard diet contains crude fat (13.5% total dietary energy), protein (27.5%) and carbohydrate (59%), and had no detectable CA derivatives, as indicated by the supplier. Mice that had been anesthetized with an inhalation of isofluorane were placed in a supine position. The mice were subcutaneously (s.c.) injected with human colon cancer HCT-116 cells (1×10^6^/0.1 ml medium) into the right flank of each BALB/C AnN-Foxn1 nude mouse. A well-localized bleb was considered to be a sign of a technically satisfactory injection.

After the inoculation, mice were divided into three subgroups (n = 6 per group). CA derivatives were given to the experimental animals by gavage once a day at a total volume 0.15 mL. The CAPE and CAPPE groups each received a daily oral dose of CA derivatives dissolved in corn oil (4% w/w) at 50 nmol/kg of BW once per day. The tumor control group received corn oil (4% w/w) once per day only. Normal mice without tumor- inoculation were used as the negative control. Tumor volume was calculated by the following formula: 0.524 L1(L2)^2^, where L1and L2 represent the long and short axis of the tumor, respectively. BW was determined once weekly. No significant differences of food intake or body weight were found in this study. At the end of the experimental period, the animals were euthanized by CO_2_ inhalation; tumor tissues were then excised, weighed, and frozen immediately. These tumor tissues were sectioned and stained with Mayer's hematoxilin– eosin (H&E) for examination by light microscopy. The remaining tissues of the liver, lung, spleen, pancreas and intestine were also excised, weighed and frozen for further experiments. Blood samples were collected from the heart in a 1-ml vacutainer tube in the presence or absence of heparin and centrifuged for 10 min at 1000 g to obtain plasma or serum, respectively.

### Histopathological and immunohistochemical staining of tumor tissues

Frozen tumor tissues were cut in 5 µm sections and immediately fixed with 4% paraformaldehyde. Sections were stained with Meyer's Hematoxylin-Eosin (H&E) for light microscopy. Negative controls did not exhibit any staining. Three hot spots were examined in a blinded manner per tumor section (high power field 200×) from six different tumors in each group. For immunohistochemical staining, frozen tissue sections were treated with 0.3% hydrogen peroxide to block the endogenous peroxide activity. Non-specific protein binding was blocked with 10% normal goat serum (NGS) for 1 hr followed by incubation with either anti-FASN or anti-PCNA primary antibodies (1∶300). Tissue sections were washed with 0.1 M phosphate buffer saline (PBS) and incubated with biotinyated immunoglobin G (1∶300 secondary antibody) at room temperature for 1 hr. Tissue sections were stained with Avidin-Biotin complex (ABC), diaminobenzidine (DAB) and hydrogen peroxide. Cell nuclei were stained with hematoxylin. Imaging was performed at 200× magnifications. Images of tumor sections were acquired on an Olympus BX-51 microscope using an Olympus DP-71 digital camera and imaging system (Olympus, Tokyo, Japan).

### Preparation of protein extraction

Human CRC HCT-116 cells were cultured in 10% FBS culture media in the presence of CAPE or CAPPE for 2 h or 24 h. Cell lysates (cytoplasmic and nuclear proteins) from colon cancer cells were prepared using the Nuclear Protein Extract Reagent Kit containing a protease inhibitor and phosphatase inhibitors according to the manufacturer's instructions. After centrifugation for 10 minutes at 12,000×g to remove cell debris, the supernatants were retained as a cytoplasmic extract. Cross contamination between nuclear and cytoplasma fractions was not detected (data not shown).

### Detection of Plasma MMP-9 by Enzyme-Linked Immunosorbent Assay (ELISA)

The MMP-9 plasma level was measured by ELISA according to the manufacturer's instructions (R&D Systems Inc.). Briefly, a 100 µL diluted plasma sample (1∶8 dilution) from each group was added to each well and analyzed. Upon completion of the ELISA process, the plate was read at 450/570 nm wavelength using a microplate reader (Tecan Inc., Mannedorf, Switzerland).

### Analysis of cellular ATP levels

Human CRC cells were cultured for 24 h in 96-well plates, each well containing 1×10^4^ cells in the presence of CAPE or CAPPE. Measurements of cellular ATP were analyzed following the manufacturer's protocol. Briefly, cell lysates were prepared using cell lysis buffer directly. Total cell lysate (100 µL) were mixed with substrate solution and vibrated to dissolve the deposits according to the manufacturer's instructions. The optical density was measured with a Synergy HT Multi-Mode Microplate Reader (BioTek, Winooski, VT).

### Western Blotting Analysis

Cellular proteins (70 µg) were fractionated on 10% SDS-PAGE, transferred to a nitrocellulose membrane, blotted with anti-phosphorylation Akt monoclonal antibody, and performed with chemiluminescence based assay. Protein phosphorylation of PDK1, phosphorylation of GSK3α, phosphorylation of GSK3β,phosphorylation of FOXO3, phosphorylation of AMPK, phosphorylation of m-TOR, PTEN, N-cadherin, PDK1, Akt, GSK3α, GSK3β, FOXO3, TSC1, TSC2, mTOR, LKB1, 14-3-3, AMPK, FASN, NF-κB (p-65), cyclin D1, Cdk4, PCNA, p21^CIP1/WAF1^, cyclin E and c-myc in the cell lysates were measured using the same procedure described above. The blots were stripped and reprobed with either β-actin or lamin A antibodies as the loading control.

### Reporter gene assay

Transfection of NF-κB response element (NF-κB-RE) plasmid was carried out in human CRC HCT-116 and SW-480 cells according to the manufacturer's instruction. Human CRC HCT-116 and SW-480 cells were then plated at a density of 2×10^5^ cells per well in 12-well plates in 2 mL of media and incubated overnight. Cells were treated with either CAPE or CAPPE at different concentrations for 24 h before the analysis of reporter gene activities. The reporter gene assay was performed by using Dual- Luciferase Reporter Assay kit. Luciferase intensities were measured using with a Synergy HT Multi-Mode Microplate Reader (BioTek, Winooski, VT).

### Statistical analysis

A quantitative methodology was used to determine whether there was any significant difference in the cell viability as well as protein expression between experimental sets and control sets of colon cancer cells. In brief, statistical analyses of the differences in cell viability among triplicate sets of the experimental conditions were performed using SYSTAT software. Confirmation of a difference in cell viability as significant requires rejection of the null hypothesis of no difference between the mean indices obtained from the replicate sets of experimental and control groups at the *P* = 0.05 level, utilizing the one way ANOVA model. The Bonferroni post hoc test was used to determine differences among the different groups.

## Results

### CA derivatives significantly inhibited the proliferation of human CRC cells *in vitro*

**Figure 2:**
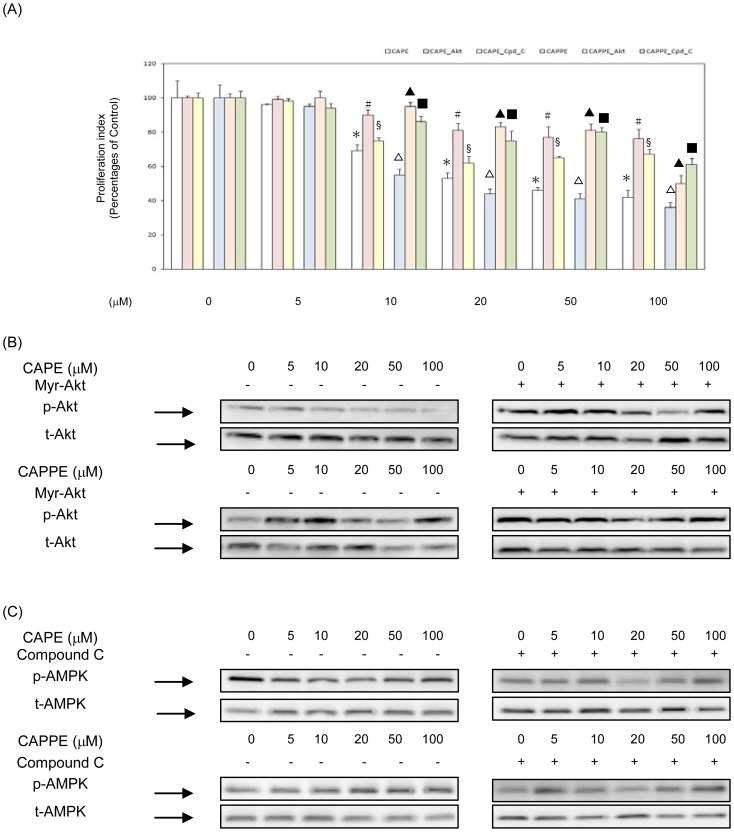
CA derivatives significantly inhibited the proliferation of human CRC HCT-116 cells *in vitro*. (A) Human CRC HCT-116 cells were cultured in RPMI-1640 medium with CAPE and CAPPE (at concentrations of 0, 5, 10, 20, 50 and 100 µM) in the presence or absence of compound C (10 µM) for 24 h. Transfections of constitutively active Akt (Myr-Akt1) and empty vector (pcDNA3) were conducted before the treatment of CA derivatives. The cell proliferation was measured by MTT assay as described in Materials and Methods. Data are the mean ± SD (standard deviation) of three independent experiments. The different symbols (??? for CAPE and ▵ for CAPPE) represent a statistically significant difference compared to the CA derivative -untreated control group in each group, respectively, at P<0.05. The different symbols (# for CAPE_Akt, § for CAPE_compound C, ▴ for CAPPE_Akt, and ▪ for CAPPE_compound C) represent a statistically significant difference compared to each corresponding CA derivative- treated control group in each dosage subgroup, respectively, at P<0.05. (B–C) Cytoplasmic proteins were prepared for Western blotting analysis using monoclonal antibodies against anti-phosphorylation Akt (S473), total-Akt, anti-phosphorylation AMPKα (T172) and total-AMPKα.

**Figure 3:**
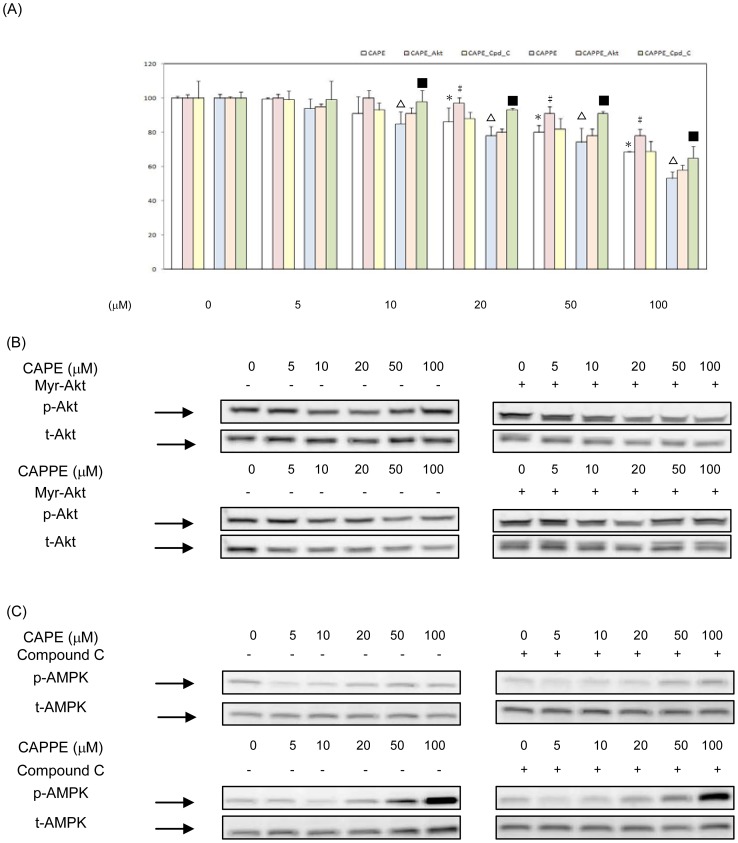
CA derivatives significantly inhibited the proliferation of human CRC SW-480 cells *in vitro*. (A) Human CRC SW-480 cells were cultured in RPMI-1640 medium with CAPE and CAPPE (at concentrations of 0, 5, 10, 20, 50 and 100 µM) in the presence or absence of compound C (10 µM) for 24 h. Transfections of constitutively active Akt (Myr-Akt1) and empty vector (pcDNA3) were conducted before the treatment of CA derivatives. The cell proliferation was measured by MTT assay as described in Materials and Methods. Data are the mean ± SD (standard deviation) of three independent experiments. The different symbols (??? for CAPE and ▵ for CAPPE) represent a statistically significant difference compared to the CA derivative -untreated control group in each group, respectively, at P<0.05. The different symbols (# for CAPE_Akt, § for CAPE_compound C, ▴ for CAPPE_Akt, and ▪ for CAPPE_compound C) represent a statistically significant difference compared to each corresponding CA derivative- treated control group in each dosage subgroup, respectively, at P<0.05. (B–C) Cytoplasmic proteins were prepared for Western blotting analysis using monoclonal antibodies against anti-phosphorylation Akt (S473), total-Akt, anti-phosphorylation AMPKα (T172) and total-AMPKα.

The inhibitory effects of CA derivatives on the proliferation of human CRC cells (HCT-116 and SW-480 cells) were investigated *in vitro*. As shown in Figure 2–3, CA derivatives (at the concentrations of 5, 10, 20, 50 and 100 µM) significantly inhibited the proliferation of human CRC HCT-116 and SW-480 cells. At the concentrations of 5, 10, 20, 50 and 100 µM, CAPE and CAPPE each significantly suppressed the proliferation of human CRC HCT-116 cells, respectively. (Inhibitory effects of CAPE: 4, 31, 47, 54, and 58%; CAPPE: 5, 45, 56, 59 and 64%) (Figure 2A). The IC50s for CAPE and CAPPE in human CRC HCT-116 cells are 44.2 µM and 32.7 µM, respectively. At the concentrations of 5, 10, 20, 50 and 100 µM, CAPE and CAPPE significantly suppressed the proliferation of human CRC SW-480 cells, respectively. (Inhibitory effects of CAPE: 0.5, 8.9, 14, 19 and 32%; CAPPE: 6, 15, 22, 26 and 47%) (Figure 3A). The IC50s for CAPE and CAPPE in human CRC SW-480 cells are 132.3 µM and 130.7 µM, respectively. These results demonstrate that CAPE and CAPPE are each able to significantly inhibit the proliferation of human CRC cells in a dose-dependent manner. CAPPE seems to inhibit the proliferation of human CRC HCT-116 cells more effectively than CAPE. For this reason, CAPE and CAPPE were selected for further study of their potential anti-cancer effects on human CRC cells. The role of signalling molecules on cell proliferation in human CRC cells treated with CA derivatives was investigated. In these cells, Akt was either over-expressed by transfection with a constitutively active Myr-Akt1 plasmid, or AMPK activity was inhibited by compound C. As shown in Figure 2A, both over-expression of Akt and suppression of AMPK activity rescued cell proliferation inhibited by CAPE or CAPPE treatments in human CRC HCT-116 cells. The effects of Akt over-expression or reduced AMPK activity on rescuing cell proliferation were less significant, however, in SW-480 cells treated with CA-derivative (Figure 3A). Expression levels of p-Akt and t-Akt proteins by the overexpression of a constitutively active form of Akt in human CRC HCT-116 and SW-480 cells were shown in Figure 2B and Figure 3B, respectively. Expression levels of p-AMPK and t-AMPK proteins by the treatment of compound C in human CRC HCT-116 and SW-480 cells were shown in Figure 2C and Figure 3C, respectively. The results suggested that CA derivatives act as chemopreventive agents against human CRC through a modulation of the PI3-K/Akt and AMPK signaling pathways

### CAPE and CAPPE each induced G_0_/G_1_ cell cycle arrest in CRC cells

**Figure 4:**
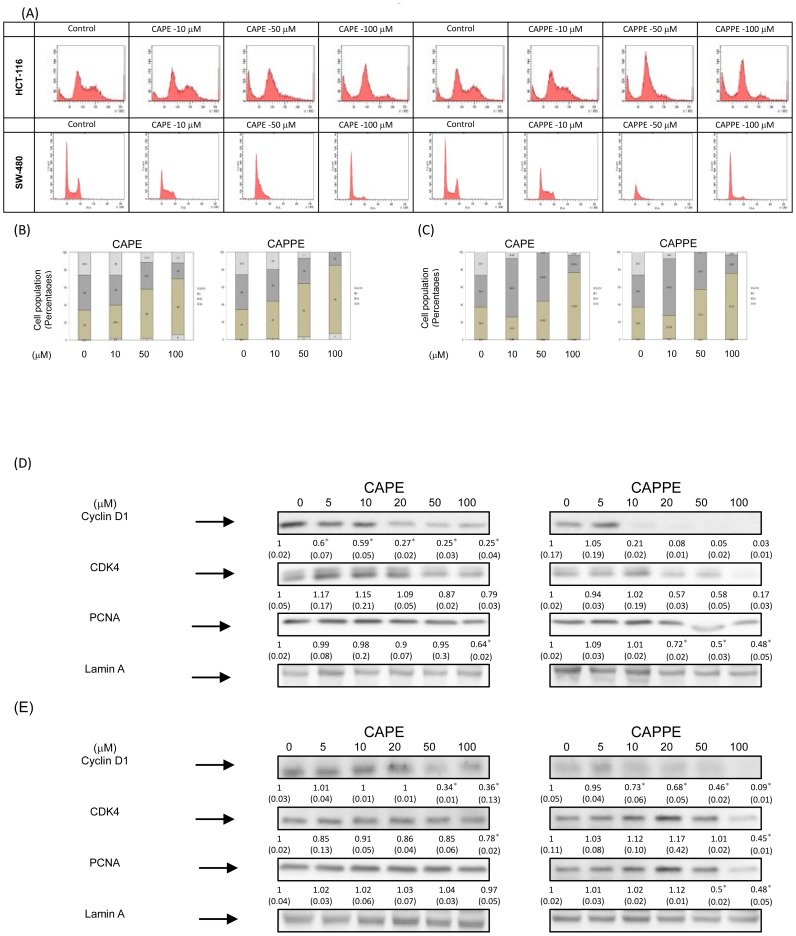
CAPE and CAPPE each induced G_0_/G_1_ cell cycle arrest in CRC cells. Human CRC cells were synchronized in RPMI-1640 medium with 0.05% FBS in tissue culture dishes overnight. To measure the distribution of the cell cycle, cell were cultured in the presence or absence of CAPE and CAPPE (0, 10, 50 and 100 µM) cultured in 10% FBS RPMI-1640 medium for another 24 h. (A) The measurement of the cell population at different cell cycle phases was performed using flow cytometry analysis, as described under Materials and Methods. The data indicate the (B) HCT-116 cell (C) SW-480 cell population percentage at different cell phases under the treatment of CAPE or CAPPE in human CRC cells. Human CRC (D) HCT-116 cells (E) SW-480 cells were treated with either CAPE or CAPPE (at concentrations of 0, 5, 10, 20, 50 and 100 µM) in 10% FBS RPMI-1640 for 24 h. Nuclear proteins were prepared for Western blotting analysis using monoclonal antibodies against cyclin D1, Cdk4, PCNA, and lamin A antibodies, as described under Materials and Methods. The levels of detection represent the amounts of cyclin D1, Cdk4 and PCNA in the nuclei of human CRC cells. The results (mean ± SD) represent the folds change of control group and are representative of three different experiments. The immunoreactive bands are noted with an arrow. The mean integrated densities of these proteins adjusted with the internal control lamin A protein are shown in bottom row. The standard deviation (SD) of each measured protein was indicated in the parenthesis. A single asterisk indicates a significant difference compared to the CAPE- or CAPPE-untreated control group, respectively (*P*<0.05).

To determine whether CA derivative-mediated suppression of cell proliferation was due to an arrest at a certain stage of the cell cycle, the effects of CAPE and CAPPE were studied further in HCT-116 and SW-480 cells. Cells treated with CAPE or CAPPE were subjected to flow cytometric analysis after their DNA was stained with PI. Histograms of the flow cytometric data are shown in Figure 4A. CAPE and CAPPE significantly induced cell cycle arrest at the G_0_/G_1_ phase in a dose-dependent manner (P<0.05). At a concentration of 50 µM, CAPE and CAPPE significantly increased cell cycle arrest of HCT-116 cells during the G_0_/G_1_ phase by up to 56% and 61%, respectively, whereas in the control group the percentage of cells in the G_0_/G_1_ phase was only 34% (Figure 4B). At a concentration of 50 µM, CAPE and CAPPE significantly increased cell cycle arrest of SW-480 cells during the G_0_/G_1_ phase by up to 44% and 57%, respectively, whereas in the control group the percentage of cells in the G_0_/G_1_ phase was only 37% (Figure 4C). These increases in G_0_/G_1_ arrest were mostly at the expense of the S and G_2_/M phase cell populations. CAPPE seems to induce G_0_/G_1_ cell cycle arrest more effectively than CAPE in human CRC cells. Thus, it is plausible that CAPE and CAPPE inhibited cell proliferation of human CRC cells through a cell cycle arrest at the G_0_/G_1_ phase.

To determine the molecular mechanisms underlying these effects, we further investigated the chemopreventive effects of CA derivatives on human CRC cells. A previous study had indicated that the cell cycle progression through G1 phase is primarily regulated by cyclinD1/Cdk4 proteins [33]. To investigate the possible inhibitory effects of CA derivatives on cell cycle regulatory proteins, HCT-116 and SW-480 cells were treated with the aforementioned concentrations of CAPE and CAPPE and the expression of nuclear proteins were measure by western blot analysis. As shown in Figure 4D (HCT-116 cells) and 4E (SW-480 cells), CAPE and CAPPE each significantly inhibited the expression of the cyclin D1 protein in a dose-dependent manner. CAPE and CAPPE also suppressed the expression of proliferating cell nuclear antigen (PCNA) protein in CRC cells. These results indicated that CAPE and CAPPE significantly induced cell cycle arrest of CRC cells at the G_0_/G_1_ phase through suppression of the nuclear cyclin D1 and PCNA proteins.

### CAPE and CAPPE inhibited the proliferation of human CRC cells through the modulation of the PI3K/Akt, AMPK and mTOR signaling pathways

**Figure 5:**
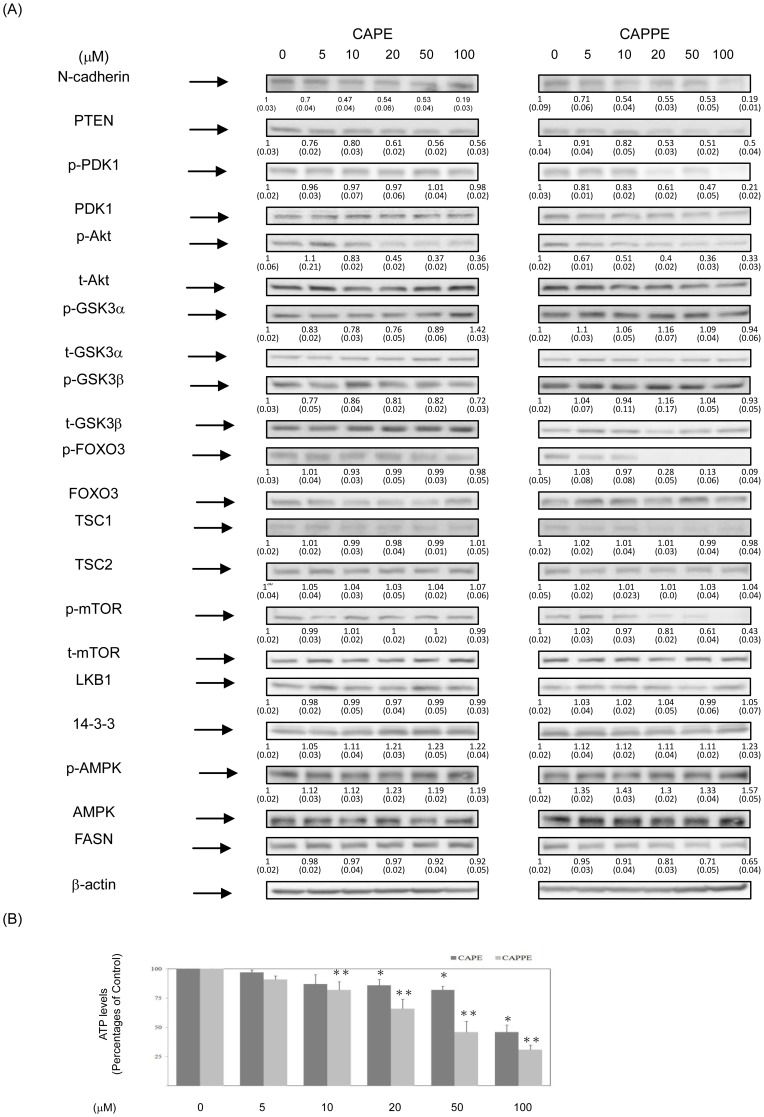
CAPE and CAPPE inhibited the proliferation of human CRC HCT-116 cells through the modulation of the PI3K/Akt, AMPK and mTOR signaling pathways. Human CRC HCT-116 cells were treated with either CAPE or CAPPE (at concentrations of 0, 5, 10, 20, 50 and 100 µM) in 10% FBS RPMI-1640 for 24 h. (A) Cytoplasmic proteins were prepared for Western blotting analysis using monoclonal antibodies against N-cadherin, PTEN, anti-phosphorylation PDK1 (S241), total-PDK1, anti-phosphorylation Akt (S473), total-Akt, anti-phosphorylation GSK3α (S21), total- GSK3α, anti-phosphorylation GSK3β (S9), total- GSK3β, anti-phosphorylation FOXO3 (T32), total- FOXO3, total- TSC1, total- TSC2, total- LKB1, total- 14-3-3, anti-phosphorylation AMPKα (T172), total-AMPKα, anti-phosphorylation m-TOR (S2448), total-m-TOR, anti-FASN and β-actin as described under Materials and Methods. The levels of detection represent the amounts of each protein in the cytoplasm of HCT-116 cells. The results (mean ± SD) represent the folds change of control group and are representative of three different experiments. The immunoreactive bands are noted with an arrow. The mean integrated densities of these proteins adjusted with the control protein are shown in bottom row. The standard deviation (SD) of each measured protein was indicated in the parenthesis. A single asterisk indicates a significant difference compared to the CAPE- or CAPPE-untreated control group, respectively (*P*<0.05). (B) The measurement of cellular ATP was performed as described under Materials and Methods. Data represent the percentage of cellular ATP levels in the CAPE- or CAPPE-treated human CRC HCT-116 cells. A single or double asterisk indicates a significant difference compared to the CAPE- or CAPPE-untreated control group, respectively (*P*<0.05).

Previous studies indicated that the PI3-K/Akt, mTOR and AMPK signaling pathways play important roles in the growth and progression of human CRC [18], [34]–[37]. To explore the molecular mechanisms by which inhibition of signaling cascades might induce cell cycle arrest, we investigated the inhibitory effects of CAPE and CAPPE on the PI3-K/Akt, mTOR and AMPK signaling pathways. As shown in Figure 5 (HCT-116 cells) and 6 (SW-480 cells), CAPE and CAPPE significantly inhibited the phosphorylation of the PDK1, Akt and mTOR signaling molecules compared to untreated control cells. Moreover, treatment with CAPE or CAPPE significantly augmented the expression of the 14-3-3 protein and the phosphorylation of the FOXO3 proteins. Previous studies showed that the upregulation of N-cadherin is associated with the progression of carcinoma cells [38]. Here, the results demonstrated that CAPPE significantly inhibited the expression of N-cadherin in CRC cells. These results suggested that CAPE and CAPPE each significantly inhibited cell proliferation and progression through the modulation of PI-3K/Akt and mTOR cascades, as well as the downstream target molecules, in HCT-116 cells.

**Figure 6:**
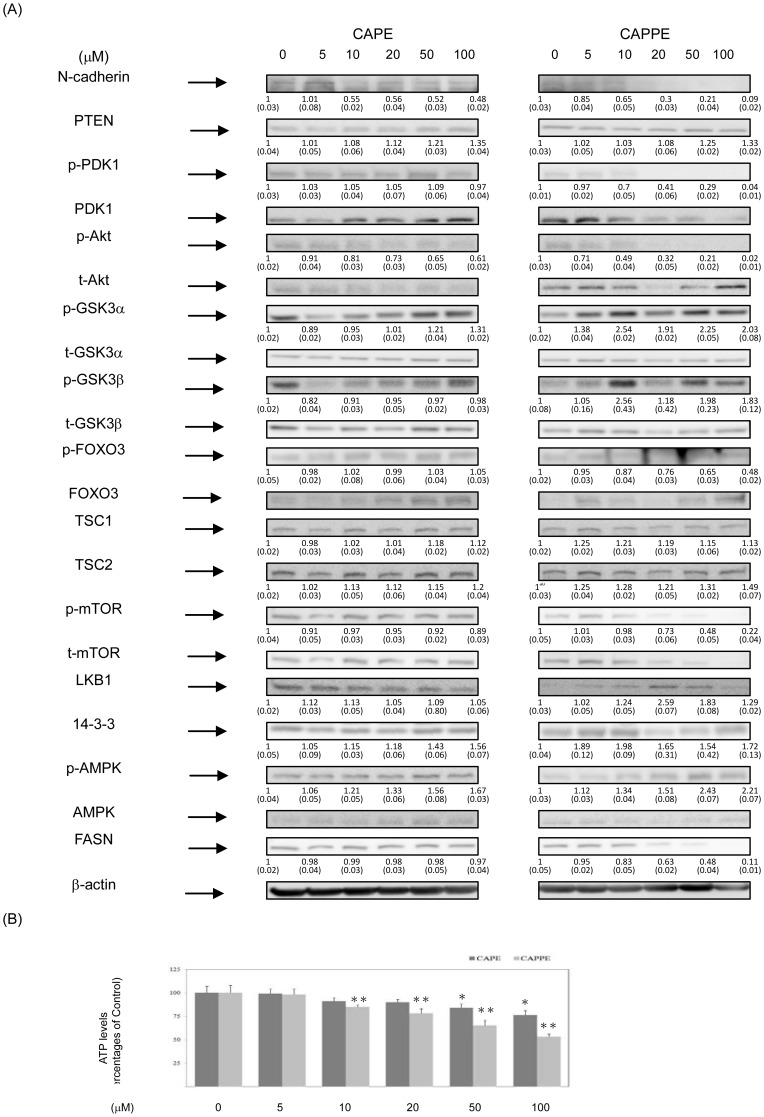
CAPE and CAPPE inhibited the proliferation of human CRC SW-480 cells through the modulation of the PI3K/Akt, AMPK and mTOR signaling pathways. Human CRC SW-480 cells were treated with either CAPE or CAPPE (at concentrations of 0, 5, 10, 20, 50 and 100 µM) in 10% FBS RPMI-1640 for 24 h. (A) Cytoplasmic proteins were prepared for Western blotting analysis using monoclonal antibodies against N-cadherin, PTEN, anti-phosphorylation PDK1 (S241), total-PDK1, anti-phosphorylation Akt (S473), total-Akt, anti-phosphorylation GSK3α (S21), total- GSK3α, anti-phosphorylation GSK3β (S9), total- GSK3β, anti-phosphorylation FOXO3 (T32), total- FOXO3, total- TSC1, total- TSC2, total- LKB1, total- 14-3-3, anti-phosphorylation AMPKα (T172), total-AMPKα, anti-phosphorylation m-TOR (S2448), total-m-TOR, anti-FASN and β-actin as described under Materials and Methods. The levels of detection represent the amounts of each protein in the cytoplasm of HCT-116 cells. The results (mean ± SD) represent the folds change of control group and are representative of three different experiments. The immunoreactive bands are noted with an arrow. The mean integrated densities of these proteins adjusted with the control protein are shown in bottom row. The standard deviation (SD) of each measured protein was indicated in the parenthesis. A single asterisk indicates a significant difference compared to the CAPE- or CAPPE-untreated control group, respectively (*P*<0.05). (B) The measurement of cellular ATP was performed as described under Materials and Methods. Data represent the percentage of cellular ATP levels in the CAPE- or CAPPE-treated human CRC SW-480 cells. A single or double asterisk indicates a significant difference compared to the CAPE- or CAPPE-untreated control group, respectively (*P*<0.05).

Recent studies suggested that the AMPK signaling pathway is involved in FASN expression and the progression of human CRC cells via crosstalk with the PI3-K/Akt signaling cascades [21]–[24]. Therefore, we further examined the effects of CAPE and CAPPE on the AMPK signaling pathway. As shown in Figure 5A and 6A, CAPE and CAPPE each significantly augmented the phosphorylation (i.e. activation) of AMPK molecule in CRC cells. Moreover, the results also showed that CAPPE- mediated activation of AMPK pathway is associated with the suppression of FASN expression (Figure 5A and 6A) and decreased ATP levels in CRC cells (Figure 5B and 6B). The results suggested that CAPE and CAPPE suppressed the expression of FASN in human CRC cells, in part through the augmentation of AMPK signaling molecules. These results further suggest that CAPPE inhibits the PI3-K/Akt, AMPK and mTOR signaling pathways in CRC cells more effectively than CAPE (Figure 5A and 6A). Moreover, the inhibitory effect of CAPPE on the cellular ATP levels is also more significant than CAPE in CRC cells (Figure 5B and 6B).

### CAPE and CAPPE inhibited the proliferation of CRC cells independently of NF-κB signaling pathway

**Figure 7:**
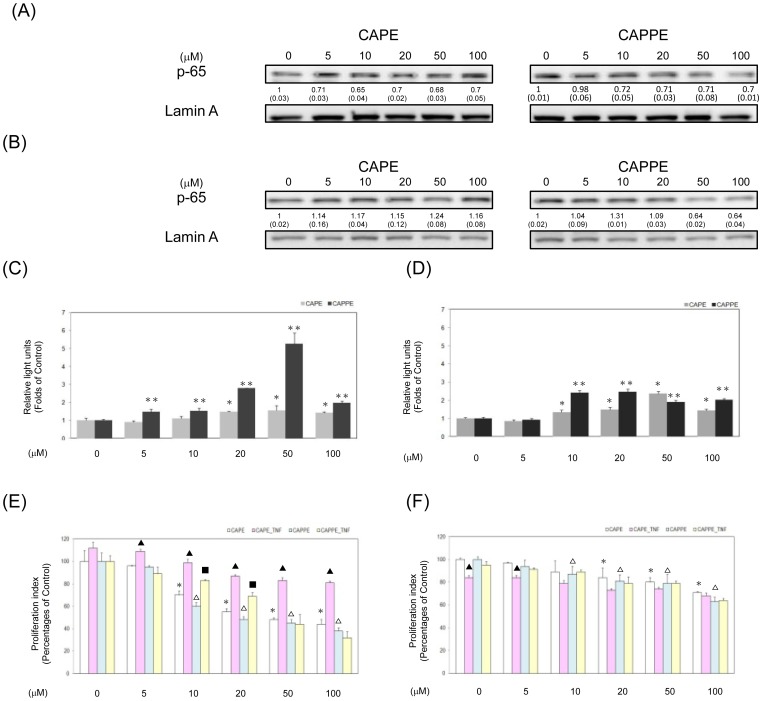
CAPE and CAPPE inhibited the proliferation of CRC cells independently of NF-κB signaling pathway. (A–B) Human CRC cells were treated with either CAPE or CAPPE (at concentrations of 0, 5, 10, 20, 50 and 100 µM) in 10% FBS RPMI-1640 for 2 h. Nuclear proteins were prepared for Western blotting analysis using monoclonal antibodies against NF-κB (p65) and lamin A as described under Materials and Methods. The levels of detection represent the amounts of each protein in the nuclei of HCT-116 cells (A) or SW-480 cells (B). The results (mean ± SD) represent the folds change of control group. The mean integrated densities of these proteins adjusted with the control protein are shown in bottom row. The standard deviation (SD) of each measured protein was indicated in the parenthesis. Human CRC HCT-116 cells (C) or SW-480 cells (D) were transfected with NF-κB-RE plasmid and then treated with either CAPE or CAPPE (at concentrations of 0, 5, 10, 20, 50 and 100 µM) in 10% FBS RPMI-1640 for 24 h. The relative light units (R.L.U) were measured by the manufacturer's instruction as described under Materials and Methods. A single or double asterisk indicates a significant difference compared to the CAPE- or CAPPE-untreated control group, respectively (*P*<0.05). Human CRC HCT-116 cells (E) or SW-480 cells (F) were cultured in RPMI-1640 medium with CAPE and CAPPE (at concentrations of 0, 5, 10, 20, 50 and 100 µM) in the presence or absence of TNF-α (1 ng/mL) for 24 h. The cell proliferation was measured by MTT assay as described in Materials and Methods. Data are the mean ± SD (standard deviation) of three independent experiments. The different symbols (??? for CAPE and ▵ for CAPPE) represent a statistically significant difference compared to the CA derivative -untreated control group in each group, respectively, at P<0.05. The different symbols (▴ for CAPE_TNF-α and ▪ for CAPPE_TNF-α) represent a statistically significant difference compared to each corresponding CA derivative- treated control group in each dosage subgroup, respectively, at P<0.05.

Previous study showed that CAPE is a well know NF-κB inhibitor in U937 cells [39]. To investigated whether CAPE and CAPPE inhibited the proliferation of human CRC cells through NF-κB pathway, the expression of NF-κB (p65; RelA) by Western blotting assay and reporter gene assay were performed in this study. The results demonstrated that CAPE or CAPPE moderately inhibited the expression of NF-κB (p65; RelA) protein in HCT-116 cells at 2 h time point (Figure 7A). Moreover, the expression of NF-κB (p65) protein was only inhibited by the treatment of CAPPE rather than CAPE in SW-480 cells at 2 h time point (Figure 7B). However, CAPE and CAPPE did not suppress the reporter gene activities of NF-κB response element (NF-κB-RE) in HCT-116 (Figure 7C) or SW-480 cells (Figure 7D) at 24 h time point. To determine whether NF-κB inhibition is important for cell proliferation, tumor necrosis factor-α (TNF-α; a NF-κB activator) was utilized in this study. The results showed that CAPE and CAPPE had differential effects on the suppression of cell growth in HCT-116 (Figure 7E) or SW-480 cells (Figure 7F) in the presence of TNF-α (1 ng/mL). These results suggested that CAPE and CAPPE mediated-suppression of cell growth was independent of NF-κB pathway in human CRC cells.

### Consumption of CAPE or CAPPE suppressed the growth of colorectal tumor in a mouse xenograft model

**Figure 8:**
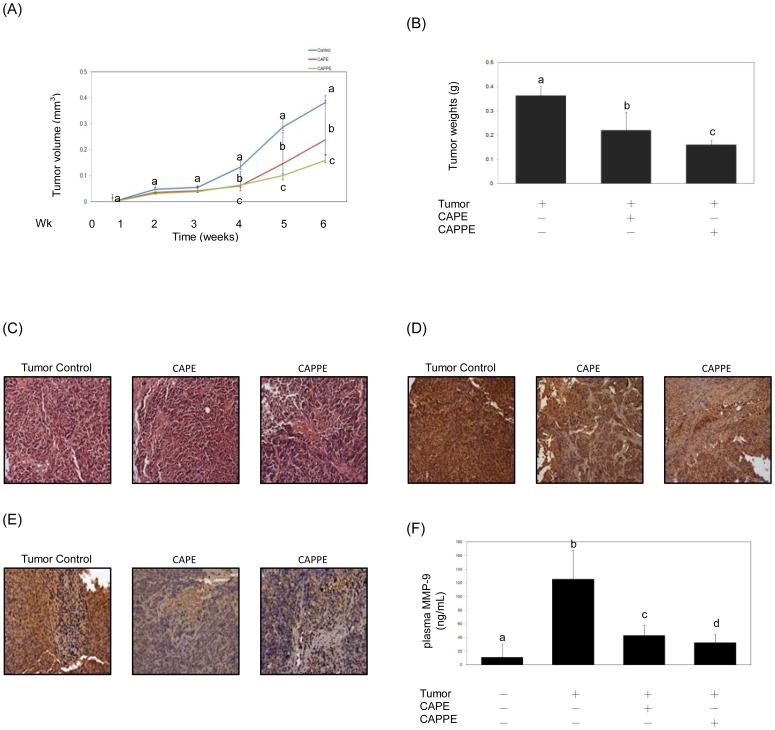
Consumption of CAPE or CAPPE suppressed the growth of colorectal tumor in a mouse xenograft model. Xenograft nude mice (n = 6 for each group) were divided into three groups (the tumor group, tumor with CAPE, tumor with CAPPE) and given CAPE or CAPPE (at a dosage of 50 nmol/kg of body weight (BW)/day) for 6 weeks. Data (mean ± SD) represent the change in the tumor volume (A) or tumor weight (B) among the tumor group (i.e. the control group), tumor with CAPE and tumor with CAPPE. The different letters at the same time point represent a statistically significant difference, (*P*<0.05). Tumor tissues were formalin-fixed, embedded in paraffin, sectioned and subjected to hematoxylin-eosin (H&E) staining (C) as described under Materials and Methods. Blue spots represent the nuclei stained with hematoxylin. The red spots represent cytoplasm stained with eosin. For immunohistochemical (IHC) staining, tumor tissues (at week 6) were frozen, sectioned and subjected to either anti- PCNA (D) or anti-FASN (E) antibodies. The intense dark brown color indicates the distribution of the PCNA or FASN proteins in HCT-116 cells stained with a monoclonal antibody. The blue area represents the localization of the cell nuclei. Imaging was documented at 200× magnification. (F) The plasma levels of MMP-9 were determined using an ELISA Kit (R&D systems). Upon completion of the ELISA process, fluorescence intensities were read using a wavelength of 450/570 nm. The results presented are representative of six different experiments and are presented as plasma MMP-9 levels. The different letters represent a significant difference in a comparison of normal mice, tumor control mice, CAPE-treat mice and CAPPE-treated mice, P<0.05.

To verify these *in vitro* findings, we further examined the respective effects of CAPE and CAPPE on the growth of human colon cancer HCT-116 cells in a mouse xenograft model. As shown in Figure 8A, consumption of CAPE and CAPPE (at dosages of 50 nmol/kg of BW per day) significantly inhibited the growth of colorectal tumors in a mouse xenograft model (P<0.05). By the end of the 6-week study period, CAPE or CAPPE significantly reduced tumor weights (P<0.05) compared to the tumor control group (Figure 8B). Histopathological staining results indicated that consumption of either CAPE or CAPPE inhibited the growth of colorectal tumor in these experimental animals (Figure 8C). Moreover, consumption of CAPE or CAPPE also suppressed the expression of malignant biomarker proteins, such as PCNA (Figure 8 D) and FASN in tumor tissues (Figure 8 E). Previous studies had suggested that the expression of MMP-9 was associated with tumor invasion and progression of CRC [11], [40]. In the current study, we investigated whether consumption of CAPE or CAPPE modulated the expression of plasma MMP-9 proteins in these experimental animals. By the end of the study, the basal MMP-9 plasma levels in the tumor-free mice were approximately 11.3 ng/mL. Mice inoculated with colon cancer HCT-116 cells had high plasma levels of MMP-9 (mean ± SD : 125.6±14 ng/mL). The consumption of CAPE or CAPPE, however, significantly decreased the MMP-9 plasma level in these tumor-bearing mice. The MMP-9 plasma levels decreased from 125.6 ng/mL in the tumor control group to 43.1 ng/mL and 32.8 ng/mL in the CAPE and CAPPE–fed groups, respectively (Figure 8F). No hepatoxicity was induced by CAPE or CAPPE at doses of 50 nmol/kg of BW in this study (data not shown). These results show that consumption of CAPE or CAPPE significantly inhibited tumor growth of CRC in a mouse xenograft model. The chemopreventive effects of CAPE and CAPPE were in part associated with the suppression of the PCNA, FASN and MMP-9 proteins in these tumor-bearing animals.

### CAPE- or CAPPE-mediated suppression of tumor growth was associated with the modulation of the PI3-K/Akt, AMPK and mTOR signaling pathways in experimental animals

**Figure 9:**
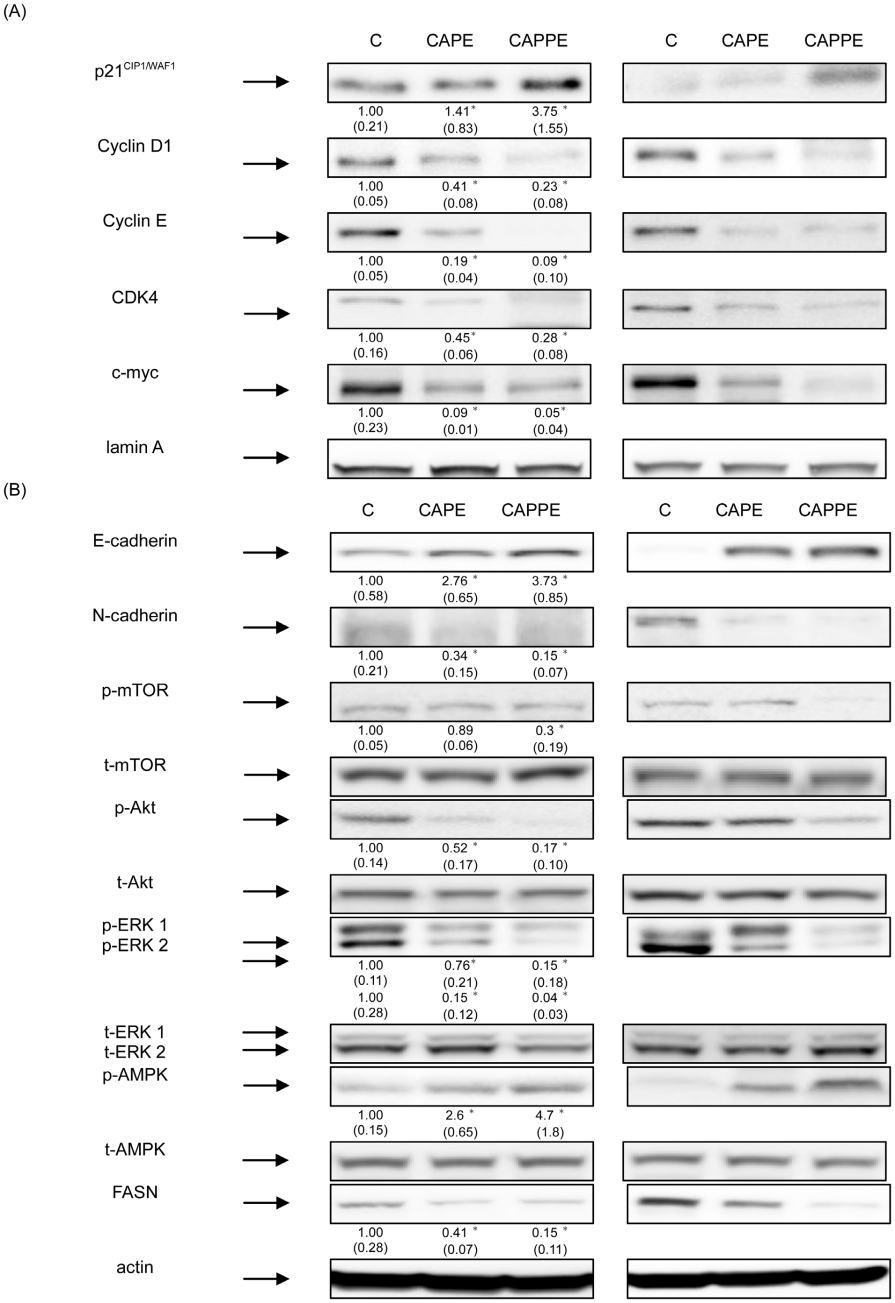
CAPE- or CAPPE-mediated suppression of tumor growth was associated with the modulation of the PI3-K/Akt, AMPK and mTOR signaling pathways in the experimental animals. (A) Nuclear proteins from tumor tissues were prepared for Western blotting analysis using monoclonal antibodies against p21^CIP1/WAF1^, cyclin D1, cyclin E, Cdk4 and c-myc as described under Materials and Methods. The results (mean ± SD) represent the folds change of control group and are representative of three different experiments. The immunoreactive bands are noted with an arrow. The levels of detection represent the amount of these proteins in the nuclei of CRC cells in the experimental animals. The mean integrated densities of these proteins are adjusted with the control protein and shown in bottom row. The standard deviation (SD) of each measured protein was indicated in the parenthesis. A single asterisk represent a statistically significant difference compared to the control group, P<0.05. (B) Cytoplasmic proteins from tumor tissues were prepared for Western blotting analysis using monoclonal antibodies against E-cadherin, N-cadherin, p-Akt, p-mTOR, p-ERK 1/2, p-AMPK, FASN and actin, as described under Materials and Methods. The results (mean ± SD) represent the folds change of control group and are representative of three different experiments. The levels of detection represent the amount of these proteins in the cytoplasm of CRC cells in the experimental animals. The mean integrated densities of these proteins are adjusted with the control protein and shown in bottom row. The standard deviation (SD) of each measured protein was indicated in the parenthesis. A single asterisk represent a statistically significant difference compared to the control group, P<0.05.

The results described above clearly show the inhibitory effects of CAPE and CAPPE on the growth of CRC cells in a mouse xenograft model. We also demonstrated the molecular mechanisms of action of the CA derivatives *in vitro*. To verify these mechanistic findings, we further examined the molecular effects of CAPE and CAPPE in these tumor-bearing mice. As shown in Figure 9A, CAPE and CAPPE consumption each significantly inhibited the expression of cyclin D1, Cdk4, cyclin E and c-myc proteins *in vivo*. Moreover, the *in vivo* chemopreventive effects of CAPE and CAPPE were associated with the upregulation of the p21^CIP1/WAF1^ protein.

It is well known that the PI3-K/Akt and MAPK/ERK signaling cascades play an important role in tumor growth and progression [4], [41]. Suppression of the PI3-K/Akt and MAPK/ERK signaling cascades leads to down-regulation of downstream target proteins such as cyclin D1/Cdk4 and a blockade of the cell cycle [4], [36], [41]–[45]. Therefore, we further investigated the inhibitory effects of CAPE and CAPPE on the PI3-K/Akt and MAPK/ERK signaling pathways. As shown in Figure 9B, consumption of CAPE or CAPPE effectively inhibited the activation of the Akt, mTOR and ERK 1/2 signaling molecules. CA derivative-mediated suppression of the Akt, mTOR and ERK 1/2 signaling cascades was associated with an up-regulation of E-cadherin as well as a suppression of N-cadherin. Moreover, CAPE and CAPPE -mediated suppression of FASN protein was associated with the augmentation of the AMPK cascade in tumor-bearing mice (Figure 9B). These results show that CAPE or CAPPE-mediated suppression of PI3-K/Akt and MAPK/ERK signaling cascades, as well as an augmentation of the AMPK signaling pathway is associated with the suppression of tumor growth at least in small laboratory animals.

## Discussion

Previous studies suggest that CAPE has potential as a chemopreventive and therapeutic agents [46]–[49]. Many studies demonstrated that CAPE could inhibit tumor angiogenesis and suppress the growth of several types of cancer [47]–[52]. The aberrant PI3K/Akt pathway has been shown to be the predominant pathway in the tumorigenesis of many types of cancer including colon cancer [53]. Studies suggested that suppression of the PI3K/Akt and integrin-mediated signaling pathways by CAPE could effectively inhibit the tumor growth [50], [54]. To date, the effects of CAPPE on the proliferation and survival of human CRC cells have not been convincingly demonstrated. In the current study, we demonstrate the inhibitory effects of CA derivatives (CAPE and CAPPE) on the proliferation of human colon cancer cells both *in vitro* and *in vivo*. The results show that CAPE and CAPPE each effectively suppressed the proliferation of human colon cancer cells in a dose-dependent manner. CAPE and CAPPE effectively suppressed the proliferation of human CRC cells through the induction of cell cycle arrest at the G_0_/G_1_ phase. Previous studies suggested that the overexpression of cell cycle-related proteins, such as D1 and Cdk4, is correlated with the proliferation of human cancer cells [33]. In this study, the results showed that CAPE or CAPPE significantly inhibited the expression of cyclin D1 protein. Recently, cyclin D1 was identified as a target of the PI3-K/Akt pathways in CRC cells [44]. We further confirmed that the molecular effects of CAPE and CAPPE were carried out through the inhibition of the PI3-K/Akt and mTOR signaling pathways in human CRC cells. Moreover, CAPE and CAPPE inhibited the expression of FASN through an augmentation of the AMPK cascade. A recent study reports that the activation of AMPK is associated with an increased cellular AMP/ATP ratio [55]. A low energy status leads to the phosphorylation (i.e. activation) of AMPK and the suppression of mTOR activity through the effect on the LKB1 protein [55]. The current study suggested than CAPPE may suppress the activity of mTOR protein in a LKB-independent manner. In contrast, CAPPE-mediated activation of the AMPK molecule was more significantly correlated with the decreased ATP levels in CRC cells. Therefore, it is probable that the respective CAPE- and CAPPE- mediated augmentation of the AMPK cascade and suppression of mTOR protein are in part associated with a decreased level of ATP in these CRC cells. There are several possible scenarios to explain why CAPPE is a more effective anti-cancer compound than CAPE. One explanation might be that CAPPE has a cell membrane solubility higher than that of CAPE. This possibility is consistent with the findings of an earlier toxicity study [56]. Previous studies demonstrated that the inhibitory effect of CA derivatives on nitric oxide (NO) production is correlated with the increasing length of the alkyl chain (i.e. CAPPE>CAPE) [56]. A recent study showed that the L-arginine -mediated NO reaction is also associated with AMPK activation [57]. These findings suggest that the upregulation of AMPK activation is dependent on the increasing length of the CA derivatives. It is to be expected, therefore, that CAPPE would be more effective in AMPK activation than CAPE. This may explain why CAPPE is a more effective regulator of AMPK activation and the suppression of cell proliferation than CAPE. The anti-proliferation effect of CAPPE could be achieved by increasing the dosage levels of CAPE (Figure 2,3). The current study also showed an inverse correlation between AMPK and mTOR activity *in vivo*. These results are consistent with AMPK -mediated downregulation of mTOR activity [22], [23]. This suggests that CAPE and CAPPE may act through this pathway as effective anti-cancer agents against human CRC cells. Moreover, the results suggested that CAPE and CAPPE mediated- suppression of cell proliferation was independent of NF-κB pathway in human CRC cells.

To verify these *in vitro* findings, we further examined the respective inhibitory effects of CAPE and CAPPE on the growth of colorectal tumor in a xenograft mouse model. As shown in Figure 8, consumption of CAPE or CAPPE significantly inhibited tumor growth *in vivo*. We also examined the actions of these bioactive compounds on multiple signaling pathways including PI3-K/Akt, MAPK/ERK and AMPK signaling cascades (Figure 9). The results demonstrated that CAPE and CAPPE also effectively induced the activation of the AMPK cascade and suppressed the activation of both the PI3-K/Akt and MAPK/ERK signaling cascades. CAPE and CAPPE further significantly inhibited the expression of FASN, cyclin D1, cyclin E, Cdk4 and c-myc proteins of tumor tissues in an *in vivo* animal study. We further examined whether the consumption of CAPE or CAPPE would help prevent tumor progression in tumor-bearing mice. The results demonstrated that CAPE or CAPPE significantly inhibited the expression of plasma MMP-9 *in vivo* (Figure 8F). These results are consistent with the *in vitro* findings.

In conclusion, this is the first demonstration of the inhibitory effects of CA derivatives (CAPE and CAPPE) on the proliferation of human colon cancer cells both *in vitro* and *in vivo*. The directional changes in protein expression produced by CAPE and CAPPE are in relevant pathways and consistent with the properties of a chemopreventive agent. Whether CAPPE is a more potent chemopreventive agent than CAPE will require further preclinical studies.

## References

[1] Siegel R, Ma J, Zou Z, Jemal A (2014) Cancer statistics, 2014. CA Cancer J Clin 64: 9–29. 24399786 10.3322/caac.21208

[2] Siegel R, Desantis C, Jemal A (2014) Colorectal cancer statistics, 2014. CA Cancer J Clin 64: 104–117. 24639052 10.3322/caac.21220

[3] Vogt PK, Kang S, Elsliger MA, Gymnopoulos M (2007) Cancer-specific mutations in phosphatidylinositol 3-kinase. Trends Biochem Sci 32: 342–349. 17561399 10.1016/j.tibs.2007.05.005

[4] Halilovic E, She QB, Ye Q, Pagliarini R, Sellers WR, et al (2010) PIK3CA mutation uncouples tumor growth and cyclin D1 regulation from MEK/ERK and mutant KRAS signaling. Cancer Res 70: 6804–6814. 20699365 10.1158/0008-5472.CAN-10-0409 PMC3178450

[5] Gustin JP, Karakas B, Weiss MB, Abukhdeir AM, Lauring J, et al (2009) Knockin of mutant PIK3CA activates multiple oncogenic pathways. Proc Natl Acad Sci U S A 106: 2835–2840. 19196980 10.1073/pnas.0813351106 PMC2636736

[6] Kang S, Bader AG, Vogt PK (2005) Phosphatidylinositol 3-kinase mutations identified in human cancer are oncogenic. Proc Natl Acad Sci U S A 102: 802–807. 15647370 10.1073/pnas.0408864102 PMC545580

[7] Wang QS, Papanikolaou A, Sabourin CL, Rosenberg DW (1998) Altered expression of cyclin D1 and cyclin-dependent kinase 4 in azoxymethane-induced mouse colon tumorigenesis. Carcinogenesis 19: 2001–2006. 9855016 10.1093/carcin/19.11.2001

[8] Wolter F, Akoglu B, Clausnitzer A, Stein J (2001) Downregulation of the cyclin D1/Cdk4 complex occurs during resveratrol-induced cell cycle arrest in colon cancer cell lines. J Nutr 131: 2197–2203. 11481417 10.1093/jn/131.8.2197

[9] Hanahan D, Weinberg RA (2011) Hallmarks of cancer: the next generation. Cell 144: 646–674. 21376230 10.1016/j.cell.2011.02.013

[10] Tang F-Y, Chiang E-PI, Sun Y-C (2008) Resveratrol inhibits heregulin-beta 1-mediated matrix metalloproteinase-9 expression and cell invasion in human breast cancer cells. Journal of Nutritional Biochemistry 19: 287–294. 17651959 10.1016/j.jnutbio.2007.03.003

[11] Aparicio T, Lehy T (1999) [Matrix metalloproteases in digestive pathology]. Gastroenterol Clin Biol 23: 330–341. 10384335

[12] Hardie DG, Scott JW, Pan DA, Hudson ER (2003) Management of cellular energy by the AMP-activated protein kinase system. FEBS Lett 546: 113–120. 12829246 10.1016/s0014-5793(03)00560-x

[13] Kahn BB, Alquier T, Carling D, Hardie DG (2005) AMP-activated protein kinase: ancient energy gauge provides clues to modern understanding of metabolism. Cell Metab 1: 15–25. 16054041 10.1016/j.cmet.2004.12.003

[14] Sato R, Goldstein JL, Brown MS (1993) Replacement of serine-871 of hamster 3-hydroxy-3-methylglutaryl-CoA reductase prevents phosphorylation by AMP-activated kinase and blocks inhibition of sterol synthesis induced by ATP depletion. Proc Natl Acad Sci U S A 90: 9261–9265. 8415689 10.1073/pnas.90.20.9261 PMC47547

[15] Fogarty S, Hardie DG (2010) Development of protein kinase activators: AMPK as a target in metabolic disorders and cancer. Biochim Biophys Acta 1804: 581–591. 19778642 10.1016/j.bbapap.2009.09.012

[16] Baba Y, Nosho K, Shima K, Meyerhardt JA, Chan AT, et al (2010) Prognostic significance of AMP-activated protein kinase expression and modifying effect of MAPK3/1 in colorectal cancer. Br J Cancer 103: 1025–1033. 20808308 10.1038/sj.bjc.6605846 PMC2965861

[17] Shackelford DB, Shaw RJ (2009) The LKB1-AMPK pathway: metabolism and growth control in tumour suppression. Nat Rev Cancer 9: 563–575. 19629071 10.1038/nrc2676 PMC2756045

[18] Liu YQ, Cheng X, Guo LX, Mao C, Chen YJ, et al (2012) Identification of an annonaceous acetogenin mimetic, AA005, as an AMPK activator and autophagy inducer in colon cancer cells. PLoS One 7: e47049. 23056575 10.1371/journal.pone.0047049 PMC3466238

[19] Menendez JA, Lupu R (2007) Fatty acid synthase and the lipogenic phenotype in cancer pathogenesis. Nat Rev Cancer 7: 763–777. 17882277 10.1038/nrc2222

[20] Kuchiba A, Morikawa T, Yamauchi M, Imamura Y, Liao X, et al (2012) Body mass index and risk of colorectal cancer according to fatty acid synthase expression in the nurses' health study. J Natl Cancer Inst 104: 415–420. 22312135 10.1093/jnci/djr542 PMC3295745

[21] Porstmann T, Griffiths B, Chung YL, Delpuech O, Griffiths JR, et al (2005) PKB/Akt induces transcription of enzymes involved in cholesterol and fatty acid biosynthesis via activation of SREBP. Oncogene 24: 6465–6481. 16007182 10.1038/sj.onc.1208802

[22] Zakikhani M, Dowling RJ, Sonenberg N, Pollak MN (2008) The effects of adiponectin and metformin on prostate and colon neoplasia involve activation of AMP-activated protein kinase. Cancer Prev Res (Phila) 1: 369–375. 19138981 10.1158/1940-6207.CAPR-08-0081

[23] Zakikhani M, Dowling R, Fantus IG, Sonenberg N, Pollak M (2006) Metformin is an AMP kinase-dependent growth inhibitor for breast cancer cells. Cancer Res 66: 10269–10273. 17062558 10.1158/0008-5472.CAN-06-1500

[24] Algire C, Amrein L, Zakikhani M, Panasci L, Pollak M (2010) Metformin blocks the stimulative effect of a high-energy diet on colon carcinoma growth in vivo and is associated with reduced expression of fatty acid synthase. Endocr Relat Cancer 17: 351–360. 20228137 10.1677/ERC-09-0252

[25] Slattery ML, Herrick JS, Lundgreen A, Fitzpatrick FA, Curtin K, et al (2010) Genetic variation in a metabolic signaling pathway and colon and rectal cancer risk: mTOR, PTEN, STK11, RPKAA1, PRKAG2, TSC1, TSC2, PI3K and Akt1. Carcinogenesis 31: 1604–1611. 20622004 10.1093/carcin/bgq142 PMC2930805

[26] Piazzon A, Vrhovsek U, Masuero D, Mattivi F, Mandoj F, et al (2012) Antioxidant activity of phenolic acids and their metabolites: synthesis and antioxidant properties of the sulfate derivatives of ferulic and caffeic acids and of the acyl glucuronide of ferulic acid. J Agric Food Chem 60: 12312–12323. 23157164 10.1021/jf304076z

[27] Nardini M, Leonardi F, Scaccini C, Virgili F (2001) Modulation of ceramide-induced NF-kappaB binding activity and apoptotic response by caffeic acid in U937 cells: comparison with other antioxidants. Free Radic Biol Med 30: 722–733. 11275472 10.1016/s0891-5849(00)00515-3

[28] Nardini M, Pisu P, Gentili V, Natella F, Di Felice M, et al (1998) Effect of caffeic acid on tert-butyl hydroperoxide-induced oxidative stress in U937. Free Radic Biol Med 25: 1098–1105. 9870564 10.1016/s0891-5849(98)00180-4

[29] Gocer H, Gulcin I (2011) Caffeic acid phenethyl ester (CAPE): correlation of structure and antioxidant properties. Int J Food Sci Nutr 62: 821–825. 21631390 10.3109/09637486.2011.585963

[30] Yilmaz HR, Uz E, Yucel N, Altuntas I, Ozcelik N (2004) Protective effect of caffeic acid phenethyl ester (CAPE) on lipid peroxidation and antioxidant enzymes in diabetic rat liver. J Biochem Mol Toxicol 18: 234–238. 15452882 10.1002/jbt.20028

[31] Chen MF, Wu CT, Chen YJ, Keng PC, Chen WC (2004) Cell killing and radiosensitization by caffeic acid phenethyl ester (CAPE) in lung cancer cells. J Radiat Res 45: 253–260. 15304968 10.1269/jrr.45.253

[32] Ribeiro U Jr, Safatle-Ribeiro AV (2007) Caffeic acid phenethyl ester (CAPE) may be a promising adjuvant treatment in gastric cancer. J Clin Gastroenterol 41: 871–873. 18090154 10.1097/MCG.0b013e31806b5938

[33] Stacey DW (2003) Cyclin D1 serves as a cell cycle regulatory switch in actively proliferating cells. Curr Opin Cell Biol 15: 158–163. 12648671 10.1016/s0955-0674(03)00008-5

[34] Vivanco I, Sawyers CL (2002) The phosphatidylinositol 3-Kinase AKT pathway in human cancer. Nat Rev Cancer 2: 489–501. 12094235 10.1038/nrc839

[35] Philp AJ, Campbell IG, Leet C, Vincan E, Rockman SP, et al (2001) The phosphatidylinositol 3′-kinase p85alpha gene is an oncogene in human ovarian and colon tumors. Cancer Res 61: 7426–7429. 11606375

[36] Ponnurangam S, Standing D, Rangarajan P, Subramaniam D (2013) Tandutinib inhibits the Akt/mTOR signaling pathway to inhibit colon cancer growth. Mol Cancer Ther 12: 598–609. 23427297 10.1158/1535-7163.MCT-12-0907 PMC4418457

[37] Wang W, Guan KL (2009) AMP-activated protein kinase and cancer. Acta Physiol (Oxf) 196: 55–63. 19243571 10.1111/j.1748-1716.2009.01980.x

[38] Tang F-Y, Pai M-H, Chiang E-PI (2012) Consumption of high-fat diet induces tumor progression and epithelial-mesenchymal transition of colorectal cancer in a mouse xenograft model. Journal of Nutritional Biochemistry 23: 1302–1313. 22221675 10.1016/j.jnutbio.2011.07.011

[39] Natarajan K, Singh S, Burke TR Jr, Grunberger D, Aggarwal BB (1996) Caffeic acid phenethyl ester is a potent and specific inhibitor of activation of nuclear transcription factor NF-kappa B. Proc Natl Acad Sci U S A 93: 9090–9095. 8799159 10.1073/pnas.93.17.9090 PMC38600

[40] Tang F-Y, Pai M-H, Kuo Y-H, Wang X-D (2012) Concomitant consumption of lycopene and fish oil inhibits tumor growth and progression in a mouse xenograft model of colon cancer. Mol Nutr Food Res 56: 1520–1531. 22961879 10.1002/mnfr.201200098

[41] Wee S, Jagani Z, Xiang KX, Loo A, Dorsch M, et al (2009) PI3K pathway activation mediates resistance to MEK inhibitors in KRAS mutant cancers. Cancer Res 69: 4286–4293. 19401449 10.1158/0008-5472.CAN-08-4765

[42] Ramana KV, Tammali R, Srivastava SK (2010) Inhibition of aldose reductase prevents growth factor-induced G1-S phase transition through the AKT/phosphoinositide 3-kinase/E2F-1 pathway in human colon cancer cells. Mol Cancer Ther 9: 813–824. 20354121 10.1158/1535-7163.MCT-09-0795 PMC2946635

[43] Gulhati P, Zaytseva YY, Valentino JD, Stevens PD, Kim JT, et al (2012) Sorafenib enhances the therapeutic efficacy of rapamycin in colorectal cancers harboring oncogenic KRAS and PIK3CA. Carcinogenesis 33: 1782–1790. 22696593 10.1093/carcin/bgs203 PMC3514899

[44] Tang F-Y, Shih C-J, Cheng L-H, Ho H-J, Chen H-J (2008) Lycopene inhibits growth of human colon cancer cells via suppression of the Akt signaling pathway. Mol Nutr Food Res 52: 646–654. 18537129 10.1002/mnfr.200700272

[45] Tang F-Y, Pai M-H, Wang X-D (2011) Consumption of Lycopene Inhibits the Growth and Progression of Colon Cancer in a Mouse Xenograft Model. J Agric Food Chem 59: 9011–9021. 21744871 10.1021/jf2017644

[46] Tolba MF, Esmat A, Al-Abd AM, Azab SS, Khalifa AE, et al (2013) Caffeic acid phenethyl ester synergistically enhances docetaxel and paclitaxel cytotoxicity in prostate cancer cells. IUBMB Life 65: 716–729. 23847086 10.1002/iub.1188

[47] Lin HP, Jiang SS, Chuu CP (2012) Caffeic acid phenethyl ester causes p21 induction, Akt signaling reduction, and growth inhibition in PC-3 human prostate cancer cells. PLoS One 7: e31286. 22347457 10.1371/journal.pone.0031286 PMC3274546

[48] Chuu CP, Lin HP, Ciaccio MF, Kokontis JM, Hause RJ Jr, et al (2012) Caffeic acid phenethyl ester suppresses the proliferation of human prostate cancer cells through inhibition of p70S6K and Akt signaling networks. Cancer Prev Res (Phila) 5: 788–797. 22562408 10.1158/1940-6207.CAPR-12-0004-T PMC4962698

[49] Kuo YY, Lin HP, Huo C, Su LC, Yang J, et al (2013) Caffeic Acid Phenethyl Ester Suppresses Proliferation and Survival of TW2.6 Human Oral Cancer Cells via Inhibition of Akt Signaling. Int J Mol Sci 14: 8801–8817. 23615471 10.3390/ijms14058801 PMC3676757

[50] Pramanik KC, Kudugunti SK, Fofaria NM, Moridani MY, Srivastava SK (2013) Caffeic acid phenethyl ester suppresses melanoma tumor growth by inhibiting PI3K/AKT/XIAP pathway. Carcinogenesis 34: 2061–2070. 23640046 10.1093/carcin/bgt154 PMC3765043

[51] Xiang D, Wang D, He Y, Xie J, Zhong Z, et al (2006) Caffeic acid phenethyl ester induces growth arrest and apoptosis of colon cancer cells via the beta-catenin/T-cell factor signaling. Anticancer Drugs 17: 753–762. 16926625 10.1097/01.cad.0000224441.01082.bb

[52] Liao HF, Chen YY, Liu JJ, Hsu ML, Shieh HJ, et al (2003) Inhibitory effect of caffeic acid phenethyl ester on angiogenesis, tumor invasion, and metastasis. J Agric Food Chem 51: 7907–7912. 14690372 10.1021/jf034729d

[53] Bates RC, Edwards NS, Burns GF, Fisher DE (2001) A CD44 survival pathway triggers chemoresistance via lyn kinase and phosphoinositide 3-kinase/Akt in colon carcinoma cells. Cancer Res 61: 5275–5283. 11431370

[54] Weyant MJ, Carothers AM, Bertagnolli ME, Bertagnolli MM (2000) Colon cancer chemopreventive drugs modulate integrin-mediated signaling pathways. Clin Cancer Res 6: 949–956. 10741720

[55] Shaw RJ, Kosmatka M, Bardeesy N, Hurley RL, Witters LA, et al (2004) The tumor suppressor LKB1 kinase directly activates AMP-activated kinase and regulates apoptosis in response to energy stress. Proc Natl Acad Sci U S A 101: 3329–3335. 14985505 10.1073/pnas.0308061100 PMC373461

[56] Uwai K, Osanai Y, Imaizumi T, Kanno S, Takeshita M, et al (2008) Inhibitory effect of the alkyl side chain of caffeic acid analogues on lipopolysaccharide-induced nitric oxide production in RAW264.7 macrophages. Bioorg Med Chem 16: 7795–7803. 18667320 10.1016/j.bmc.2008.07.006

[57] Mohan S, Patel H, Bolinaga J, Soekamto N (2013) AMP-activated protein kinase regulates L-arginine mediated cellular responses. Nutr Metab (Lond) 10: 40. 23718875 10.1186/1743-7075-10-40 PMC3680329

